# Glycemic Indices and Polysomnographic Parameters in an Iranian Population Referred to a Sleep Clinic

**DOI:** 10.1055/s-0046-1829079

**Published:** 2026-10-19

**Authors:** Marjan Zahernia, Zahra Banafsheh Alemohammad, Arezu Najafi

**Affiliations:** 1 Occupational Sleep Research Center, Baharloo Hospital, Tehran University of Medical Sciences, Tehran, Iran; 2 Sleep Breathing Disorders Research Center, Tehran University of Medical Sciences, Tehran, Iran

**Keywords:** polysomnography, hypoxia, fasting blood glucose, HbA1c

## Abstract

**Introduction:**

Diabetes remains a significant global health burden, with sleep breathing disorders increasingly implicated in its development and progression. In this study, we aimed to assess the relationship between glycemic indices and polysomnography (PSG) parameters among patients referred to a sleep clinic in Iran.

**Methods:**

We retrospectively studied adults at the Baharloo Hospital sleep clinic who completed overnight PSG and a recent metabolic panel, as well as validated questionnaires on sleepiness, obstructive sleep apnea (OSA) risk, and insomnia, between March 2024 and March 2025. We examined associations using Pearson correlations and adjusted linear regression models.

**Results:**

A total of 409 participants (115 females) were included. Correlation analyses revealed that higher HbA1c and fasting blood glucose (FBS) were associated with worse nocturnal oxygenation profiles, with HbA1c showing a more selective pattern. Moreover, after adjusting for confounding factors, higher HbA1c was independently associated with lower mean nocturnal oxygen saturation (beta = -0.159, p = 0.013) and lower non-REM oxygen saturation (beta = −0.179, p = 0.007). Meanwhile, higher FBS was independently associated with lower average oxygen saturation during wake (beta = −0.688, p = 0.018) and during REM sleep (beta = −0.890, p = 0.001).

**Conclusion:**

Our study highlights the relationship between glycemic measures and sleep parameters in a relatively large population of Iranian sleep clinic patients. More studies are needed to unravel the association between diabetes and sleep breathing disorders to identify their shared mechanisms.

## Introduction

Diabetes is one of the most common metabolic disorders, characterized by elevated blood glucose levels due to insulin resistance, insufficient insulin secretion, or both. Recent estimates have suggested that the prevalence of diabetes will increase to 643 million by 2030 and 783 million by 2045, posing significant healthcare and economic challenges to governments worldwide. 1 This burden is mainly attributed to the development of complications, such as diabetic kidney disease, retinopathy, and peripheral neuropathy. 2 The modern world promotes poor diet quality and a sedentary lifestyle, making individuals vulnerable to developing diabetes and other metabolic diseases. 3 In recent years, growing attention has been paid to the relationship between diabetes and sleep, a fundamental component of lifestyle 4

Poor sleep habits and sleep disorders have been reported to be common among adults with diabetes. 5 Based on observational studies, habitual short sleep duration, shift work, low-quality sleep, and long evening napping have all been associated with higher risks of developing type 2 diabetes. 6 7 8 All these findings imply further investigation, especially now that modern lifestyles are involved with longer working hours and technological entertainment activities, negatively affecting sleep. 9 Sleep disturbances have also been associated with higher systemic inflammation markers and an increasing incidence of psychiatric disorders, obesity, and hypertension, all correlated with diabetes pathophysiology. 10 11 12

Based on the evidence summarized above, further studies are needed to assess the associations between sleep and diabetes. The present study aimed to investigate the associations between glycemic indices and polysomnographic (PSG) parameters — a gold-standard measure of sleep characteristics — among patients referred to a sleep clinic in Iran. The findings of this study may facilitate early identification of patients at risk for both sleep disorders and diabetes, improve disease management, and reduce complications. These findings may support the development of risk-assessment tools and help reduce the incidence of diabetes among individuals with sleep disorders.

## Materials and Methods

### Study Design and Participants

This retrospective, observational study was conducted in a sleep clinic in Tehran, Iran. The protocol of this study was approved by the Ethics Committee of Tehran University of Medical Sciences (IR.TUMS.MEDICINE.REC.1403.030). All study procedures adhered to the principles of the Declaration of Helsinki, 13 and all participants provided written informed consent prior to enrollment.

Electronic health records of patients referred to the sleep clinic who underwent overnight PSG between March 2024 and March 2025 were reviewed. And had a complete metabolic panel test in the previous two months, including fasting blood sugar (FBS), HbA1c, triglycerides, high-density lipoprotein (HDL), low-density lipoprotein (LDL), and total cholesterol. All patients in the sleep clinic are also asked to complete sleep-related questionnaires regarding daytime sleepiness, risk for obstructive sleep apnea (OSA), and insomnia. Participants with incomplete data, sleep duration of less than three hours during PSG, or pre-existing respiratory disorders, inflammatory conditions, or malignancies were excluded. Moreover, we excluded those who were shift workers.

### Measurements

#### Epworth Sleepiness Scale (ESS)

The ESS questionnaire assesses daytime sleepiness through eight items. 14 The Persian version of this questionnaire has been translated and validated by Sadeghniiat Haghighi et al., showing excellent internal consistency and test-retest reliability (Cronbach's α: 0.82 and intraclass correlation coefficient: 0.81). 15

#### STOP-BANG

The STOP-BANG questionnaire combines the STOP questionnaire (snoring, tiredness during daytime, observed apnea, and high blood pressure) with body mass index (BMI), age, neck circumference, and sex to screen for OSA risk. 16 It consists of eight dichotomous (yes/no) questions; scores above 5 indicate high risk for OSA. In Iran, Sadeghniiat Haghighi et al. translated the original questionnaire and developed the Persian version, with an intraclass correlation coefficient of 0.88. 17

#### Insomnia Severity Index (ISI)

ISI is a seven-item questionnaire subjectively evaluating insomnia. Perceived severity of each insomnia-related item is rated on a 0–4 scale, yielding a total score from 0 to 28. 18 In Iran, Yazdi et al. translated the original items and validated their reliability and validity. Their results showed a Cronbach's α coefficient above 0.8 and an intraclass correlation coefficient of above 0.7 in two groups of people with and without chronic insomnia. 19

#### Polysomnography (PSG)

PSG is the gold-standard measurement test to diagnose, confirm, and detect the severity of sleep apnea and other sleep-related disorders. 20 All participants underwent a full session of overnight PSG, and the results were analyzed by a trained sleep medicine specialist according to the American Academy of Sleep Medicine (AASM) version 3 (2023). 21 In this study, we collected the following PSG-derived measures: mean oxygen saturation, lowest oxygen saturation, saturation below 90% percentage, and average oxygen saturation during wake, average oxygen saturation during REM, average oxygen saturation during non-REM sleep, and Apnea-Hypopnea Index (AHI).

### Statistical Analysis

We reported continuous variables as mean and standard deviation (SD), and categorical variables as frequencies and percentages. To compare participant characteristics, chi-square and independent t-tests were used. Pearson correlation analyses and multivariable linear regression analyses, adjusting for potential confounders, were used to examine associations between metabolic laboratory data and sleep-related measures. All analyses were performed using SPSS Statistics, version 30 (IBM Corp., Armonk, NY, USA). Statistical significance was set at p < 0.05.

## Results

### Characteristic Data of Participants

A total of 409 participants (115 females) were included in the study. The characteristic data for all participants are presented in Table 1 . Females were older than males (mean age: 51.84 vs. 45.09 years; p < 0.001). Females had significantly higher BMI and lower STOP-BANG scores (p = 0.003 and p = 0.017, respectively).

**Table 1:** Baseline Characteristics of Participants by Sex

Variable		*P* -value
Age (Years)	46.99 ± 13.42	< 0.001*
Female	51.84 ± 11.05	
Male	45.09 ± 13.80	
BMI	30.06 ± 6.06	0.003*
Female	31.47 ± 6.91	
Male	29.50 ± 5.60	
ESS	9.59 ± 5.73	0.001*
Female	8.18 ± 4.63	
Male	10.11 ± 6.01	
STOP-BANG	4.37 ± 3.59	0.017*
Female	3.66 ± 1.89	
Male	4.64 ± 4.02	
ISI	13.39 ± 7.25	0.108
Female	14.32 ± 5.92	
Male	13.02 ± 7.68	
Mean Oxygen Saturation	90.00 ± 6.85	0.674
Female	89.77 ± 5.17	
Male	90.09 ± 7.41	
Lowest Oxygen Saturation	76.78 ± 15.94	0.936
Female	76.83 ± 16.28	
Male	76.66 ± 15.17	
Time with SpO2 < 90% (CT90%)	26.25 ± 32.55	0.227
Female	31.05 ± 31.43	
Male	24.00 ± 32.98	
Average Oxygen Saturation During Wake	90.50 ± 6.20	0.784
Female	90.37 ± 5.79	
Male	90.55 ± 6.37	
Average Oxygen Saturation During REM Sleep	89.41 ± 7.49	0.256
Female	88.72 ± 7.85	
Male	89.68 ± 7.33	
Average Oxygen Saturation During Non-REM Sleep	90.08 ± 7.98	0.922
Female	90.14 ± 4.92	
Male	90.05 ± 8.91	
AHI	40.46 ± 34.11	0.758
Female	39.63 ± 36.58	
Male	40.79 ± 33.15	
Pre-diagnosed hypertension (positive/negative)	44 (10.76%)/365 (89.24%)	0.017*
Female	19 (16.52%)/96 (83.48%)	
Male	25 (8.50%)/269 (91.50%)	
Pre-diagnosed diabetes (positive/negative)	45 (11.00%)/364 (89.00%)	0.034*
Female	19 (16.52%)/96 (83.48%)	
Male	26 (8.84%)/268 (91.16%)	
FBS	104.96 ± 27.40	0.062
Female	103.14 ± 24.33	
Male	109.62 ± 33.69	
HbA1c	6.39 ± 2.01	0.783
Female	6.26 ± 1.27	
Male	6.44 ± 2.24	

**Abbreviations:** AHI, Apnea-Hypopnea Index; BMI, Body mass index; ESS, Epworth Sleepiness Scale; F/M, Female-to-male ratio; FBS, Fasting blood sugar; ISI, Insomnia Severity Index; REM Sleep, Rapid-eye-movement sleep.

Chi-square test was conducted to compare the prevalence of hypertension and diabetes in the two groups; independent *t* -tests were applied for other variables. P-values less than 0.05 are shown with *, indicating a statistically significant difference.

### Correlations of Glycemic Measures and Sleep Parameters

Higher FBS levels were associated with worse nocturnal oxygenation profiles; FBS showed significant inverse correlations with mean oxygen saturation, lowest oxygen saturation, and average oxygen saturation during wake, REM sleep, and non-REM sleep. It also showed positive correlations with the percentage of time with oxygen saturation below 90% and with AHI. In contrast, HbA1c demonstrated a similar but more selective pattern. It had stronger inverse associations with mean oxygen saturation and average oxygen saturation during wake and REM, a positive association with time spent below 90% saturation, and no significant relationship with lowest oxygen saturation, non-REM oxygen saturation, or AHI in this sample. The details of correlation analyses are presented in Table 2 .

**Table 2:** Correlation of glycemic measures (FBS and HbA1c) with sleep parameters

		Mean Oxygen Saturation	Lowest Oxygen Saturation	Saturation Below 90 Percentage	Average Oxygen Saturation During Wake	Average Oxygen Saturation During REM Sleep	Average Oxygen Saturation During Non-REM Sleep	AHI
FBS	Pearson Correlation	−0.200	−0.207	0.323	−0.224	−0.275	−0.168	0.192
	P-value	0.000*	0.000*	0.000*	0.000*	0.000*	0.001*	0.000*
HbA1c	Pearson Correlation	−0.589	−0.247	0.527	−0.451	−0.391	−0.254	0.247
	P-value	0.000*	0.167	0.003*	0.002*	0.009*	0.085	0.094

**Abbreviations:** AHI, Apnea-Hypopnea Index; FBS, Fasting blood sugar; REM Sleep, Rapid-eye-movement sleep.

### Multivariable Regression Analyses

Adjusting for sex, age, hypertension status, HDL, LDL, triglycerides, BMI, ESS, ISI, and AHI, glycemic measures showed selective associations with sleep oxygenation. In these regression analyses, glycemic measures were set as dependent variables and sleep oxygenation measures as independent variables. Higher HbA1c was independently associated with lower mean nocturnal oxygen saturation (beta = -0.159, SE = 0.060, p = 0.013) and lower non-REM oxygen saturation (beta = -0.179, SE = 0.061, p = 0.007), with no significant associations for lowest oxygen saturation, time spent below 90% saturation, wake oxygen saturation, or REM oxygen saturation. Higher FBS was independently associated with lower average oxygen saturation during wake (beta = -0.688, SE = 0.289, p = 0.018) and during REM sleep (beta = -0.890, SE = 0.274, p = 0.001), while associations with mean oxygen saturation, lowest oxygen saturation, time spent below 90% saturation, and non-REM oxygen saturation were not significant. The details of all regression analyses are shown in Table 3 . Fig. 1 provides a visual summary of the regression coefficients and their 95% confidence intervals for the associations between oxygen saturation parameters and glycemic indices.

**Table 3:** Multivariable Regression Models for the Association between Glycemic Variables and Sleep Oxygenation Parameters

Glycemic measure	Sleep parameter	Estimate - (STD-error)	*P* -value
**HbA1c**	Mean Oxygen Saturation	-0.159 (0.060)	0.013*
	Lowest Oxygen Saturation	0.002 (0.028)	0.937
	Saturation Below 90 Percentage	0.034 (0.029)	0.272
	Average Oxygen Saturation During Wake	-0.096 (0.056)	0.097
	Average Oxygen Saturation During REM Sleep	-0.087 (0.048)	0.080
	Average Oxygen Saturation During Non-REM Sleep	-0.179 (0.061)	0.007*
**FBS**	Mean Oxygen Saturation	-0.355 (0.224)	0.114
	Lowest Oxygen Saturation	-0.145 (0.128)	0.259
	Saturation Below 90 Percentage	0.102 (0.112)	0.364
	Average Oxygen Saturation During Wake	-0.688 (0.289)	0.018*
	Average Oxygen Saturation During REM Sleep	-0.890 (0.274)	0.001*
	Average Oxygen Saturation During Non-REM Sleep	-0.339 (0.229)	0.139

**Abbreviations:** FBS, Fasting blood sugar; REM Sleep, Rapid-eye-movement sleep; STD-error, Standard error.

Note that in these multivariable regression analyses, glycemic measures were the dependent variables, and sleep parameters, along with other covariates, were considered independent variables.

**Fig. 1:**
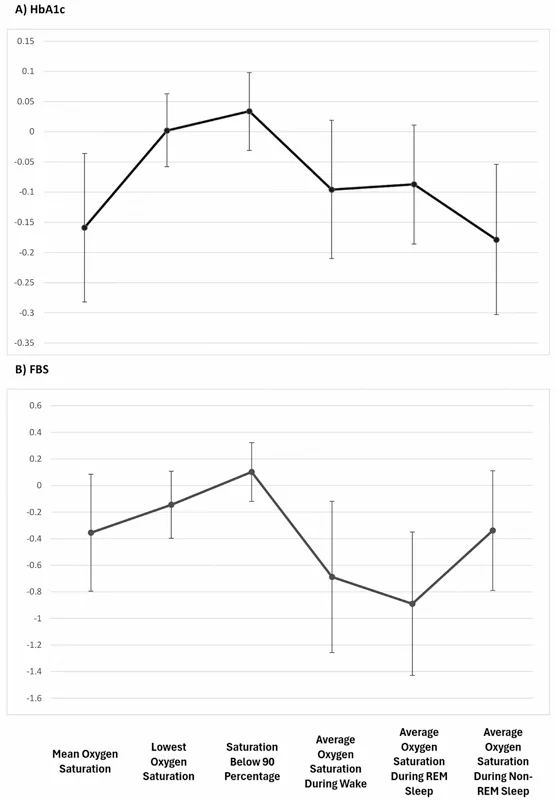
**Associations between oxygen saturation parameters and (A) HbA1c and (B) FBS in multivariable linear regression models.** Points represent adjusted unstandardized regression coefficients (B), and vertical error bars represent 95% confidence intervals. **Abbreviations:** FBS, Fasting blood sugar; REM Sleep, Rapid-eye-movement sleep.

## Discussion

Data were collected from 409 patients with a complete metabolic panel, referred to a university-affiliated sleep clinic in Iran over one year. The present results link dysglycemia to impaired nocturnal oxygenation. On the one hand, FBS showed inverse correlations with mean and lowest oxygen saturation, as well as average oxygen saturation during wake, REM sleep, and non-REM sleep, while exhibiting positive correlations with the percentage of time spent below 90% saturation and with the total AHI index. On the other hand, HbA1c showed inverse associations with mean oxygen saturation and average oxygen saturation during wake and REM, and a positive association with time spent below 90% saturation. In multivariable models adjusting for demographics, lipid biomarkers, BMI, excessive daytime sleepiness, hypertension status, and insomnia, HbA1c was linked with lower mean and non-REM oxygen saturation, while FBS was linked with lower wake and REM oxygen saturation.

Our findings indicate a significant relationship between disrupted glycemic measures and PSG parameters related to oxygenation and apnea. Previous studies confirm the relationship between OSA and diabetes; in a large prospective study by Zhang et al., 22 2,603 participants underwent PSG and diabetes assessments; those with diabetes had a higher baseline AHI and larger increases in AHI and obstructive apnea index over five years. Basille et al.23 evaluated 46 patients with type 1 diabetes. Moderate-to-severe sleep-disordered breathing was associated with higher BMI and longer diabetes duration; the present study included BMI in the regression models but lacked data on disease duration.

Poor overnight oxygenation and a diagnosis of OSA can be a risk factor for incident diabetes, too. In a large longitudinal study by Wojeck et al., 24 650 individuals without baseline diabetes underwent PSG and were followed for 8 to 10 years. Time spent with oxygen saturation below 90%, respiratory arousals, and total arousals were associated with an increased risk of diabetes. Increases in mean nocturnal oxygen saturation were also associated with decreased incidence of diabetes and prediabetes. These findings could provide complementary insights into the relationship between sleep disorders and diabetes.

Possible mechanisms underlying the relationship between diabetes and sleep breathing disorders, such as OSA, remain to be elucidated. Distinct patterns were found in the associations between glycemic factors and sleep stages. Previous studies have indicated a relationship between sleep-disordered breathing during REM sleep and impaired glucose metabolism; apnea during REM sleep has been reported to abolish the physiological decrease in blood glucose levels that naturally occurs during this stage. 25 However, the exact mechanism is not known. In general, tissue hypoxia can contribute to insulin resistance and a metabolic switch, which, in turn, can decrease oxidative phosphorylation and glucose storage while enhancing glycolysis. Short-term hypoxic exposure has been linked to increased hypothalamic-pituitary-adrenal (HPA) axis activity and elevated cortisol, which are associated with higher glucose levels. 26 Moreover, there is evidence of endothelial dysfunction in this regard. Hypoxia can contribute to the development of endothelial dysfunction, a feature present not only in diabetes but also in many cardiovascular comorbidities and complications. 27 More studies are needed to unravel the shared mechanisms of diabetes and hypoxia. Clarifying these intersecting pathways — encompassing inflammation, oxidative stress, neurohormonal activation, endothelial injury, and metabolic reprogramming — will be important for identifying therapeutic targets that can improve glycemic control and sleep disorders while mitigating cardiometabolic complications. 28

The findings of our study should be interpreted in light of some limitations. First, the study was limited to a single sleep clinic in Tehran, limiting generalizability to similar patient populations. Second, the cross-sectional nature of our study can only detect associations, not causations. The exact pathophysiological mechanisms linking diabetes and OSA remain to be elucidated — specifically, how diabetes and metabolic syndrome affect sleep oxygenation, and how sleep-disordered breathing in turn affects metabolic regulation, and a cross-sectional, single-center study such as ours could not lead to reliable findings in this regard. Third, we did not have sufficient data regarding diabetes duration, medication use history, treatment adherence, smoking status, physical activity, and measures of central obesity. These variables may be important confounders in the relationship between glycemic measures and OSA-related factors. Although we adjusted for BMI and hypertension in multivariable analyses, residual confounding and selection bias inherent to a sleep clinic-based population remain possible. Also, the relatively small number of participants in some subgroups (e.g., those with diabetes mellitus) and subjective assessment of past medical history limited our ability to perform adequately powered subgroup analyses based on pre-existing disorders. Therefore, potential differences in associations according to diabetes or obesity status could not be reliably assessed. Finally, metabolic laboratory measurements were obtained up to two months before PSG. While HbA1c reflects relatively stable long-term glycemic control, FBS is more susceptible to short-term metabolic fluctuations. As a result, the temporal discordance between FBS measurement and PSG acquisition may have introduced measurement variability and reduced the precision of associations analyzed.

In conclusion, our study highlights the relationship between glycemic measures (FBS and HbA1c) and sleep parameters in a relatively large group of Iranian patients referred to a sleep clinic over one year. These findings, along with prior longitudinal evidence, suggest that oxygenation metrics may serve as potential indicators of metabolic risk. However, it should be noted that the relatively high mean AHI in our cohort suggests that the study population was largely composed of patients with more severe forms of sleep-disordered breathing. Therefore, our findings may primarily reflect a severe OSA phenotype and should be generalized cautiously to individuals with mild-to-moderate OSA or to community-based populations. Future research should prioritize multicenter, adequately powered prospective studies that stratify by disease duration, medication use and adherence. Future studies should employ causal frameworks with repeated measures to track trajectories of glycemia and oxygenation and test the effects of therapeutic interventions, such as OSA treatment, weight management, and glycemic control, in affected individuals.
